# Draft genome sequence of *Enterococcus raffinosus* strain MEBT1 isolated from the feces of children with diarrhea

**DOI:** 10.1128/mra.01415-25

**Published:** 2026-03-16

**Authors:** Md Ashikuzzaman Antor, Abu Reza, Mahadi Hasan Sojol, Md. Monir Hossen, Md. Minhajul Abedin, Loby Mahmud, Nilufa Akhter Banu, Mohammad Abu Hena Mostofa Jamal, Md. Rezuanul Islam

**Affiliations:** 1 Department of Biotechnology and Genetic Engineering, Faculty of Biological Sciences, Islamic University 247297, Kushtia, Bangladesh; 2 Laboratory of Medical and Environmental Biotechnology, Islamic University 247297, Kushtia, Bangladesh; 3 Department of Genetic Engineering and Biotechnology, Faculty of Health and Life Sciences, Daffodil International University 130058, Dhaka, Bangladesh; Loyola University Chicago, Chicago, Illinois, USA

**Keywords:** whole-genome sequence, *Enterococcus raffinosus* strain MEBT1, feces, pathogen, children with diarrhea

## Abstract

This study presents the draft genome sequence of *Enterococcus raffinosus* strain MEBT1, isolated from the feces of children with diarrhea in Bangladesh on 1 January 2024. The assembled genome comprises 4,423,302 bp distributed across 162 contigs, with an average GC content of 39.5%, and encodes 4,313 predicted protein-coding sequences.

## ANNOUNCEMENT

*Enterococcus raffinosus* is a rare enterococcal species distinct from the clinically predominant *Enterococcus faecalis* and *Enterococcus faecium* and is infrequently associated with human infections (1). *E. raffinosus* isolated from human clinical specimens, including feces, exhibits multidrug resistance and notable virulence (2–4). This report illustrates the draft genome assembly of *E. raffinosus* strain MEBT1.

A fecal sample was collected on 1 January 2024 from the 250-bed General Hospital, Kushtia, Bangladesh. The sample was spread onto Schaedler agar (HiMedia, India) and incubated at 37°C overnight. Discrete colonies were subcultured in Schaedler broth to obtain a pure isolate, and a single colony was grown overnight at 37°C with 120 rpm agitation. One milliliter of culture (one passage) was used for genomic DNA extraction. Genomic DNA was extracted using the Wizard HMW DNA Extraction Kit following the manufacturer’s instructions. After cell lysis, the lysate was centrifuged at 14,000 rpm for 5 min to eliminate debris, and genomic DNA was purified from the supernatant using the Wizard Genomic DNA Purification Kit per manufacturer’s instructions. The quality and concentration of genomic DNA were evaluated using a NanoDrop spectrophotometer and a Qubit 4.0 fluorometer, with the Qubit working solution prepared at a 1:200 reagent-to-buffer ratio (5). Whole-genome sequencing was subsequently performed at the Genomic Center, icddr’b, Mohakhali, Dhaka-1212, Bangladesh. Genomic DNA fragments (≈200-300 bp) were size-selected with AMPure XP beads, and libraries were prepared using the Illumina DNA Prep Kit with adapter ligation on the epMotion 5075 System. Sequencing was performed on the Illumina MiSeq platform, producing 2 × 150 bp paired-end reads.

Raw sequencing reads were demultiplexed and converted to FASTQ using Illumina bcl2fastq v2.20.0 with default settings. Paired-end reads were quality-checked with FastQC v0.12.1 and used for downstream analyses (6). Trimmomatic v0.36 was used to trim the paired-end sequencing reads, with a minimum read length cutoff of 20 bp (7). Genome assembly was performed using SPAdes v3.15.5+galaxy2 and BV-BRC with default parameters (8, 9). The draft assembly was polished using Pilon v1.24 (10), and genome annotation was performed with NCBI Prokaryotic Genome Annotation Pipeline (PGAP v.6.9) employing the best-placed reference protein set and GeneMarkS-2+ v6.9 (11). Bacterial identification was confirmed by 16S rRNA gene sequencing using the Sanger dideoxy method (12). Taxonomic identification of the isolate was performed using the Type (Strain) Genome Server (13). Both the 16S rRNA gene and whole-genome phylogenetic analysis confirmed that the isolate belongs to *E. raffinosus*, which was designated strain MEBT1. Unless specified otherwise, default parameters were used for all software tools throughout this study.

The draft genome of *E. raffinosus* strain MEBT1 is 4,423,302 bp in 162 contigs, with 96.33× coverage and 39.5% GC content, consistent with BV-BRC data (39.29% GC, 181 contigs), reflecting typical genome assembly metrics. A total of 4,481 genes, including 4,313 protein-coding sequences, were predicted. Key genomic features are summarized in Table 1.

**TABLE 1:** Genomic features of *Enterococcus raffinosus* strain MEBT1

Genomic feature	Value	
Description of source		
Location	Kushtia, Bangladesh	
Time	2024	
Type	Human feces	
Sequencing summary		
Coverage	96.33×	
Number of raw reads	1,045,813	
Total bases	504.1 M	
Size	286.2 MB	
Assembly report		
Tool used	SPAdes v. Galaxy	BV-BRC
Contigs	162	181
GC content	39.5%	39.29%
Contig L50	50	15
Genome size	4.4 Mb	4,423,302 bp
Contig N50	110.6 kb	110,642 bp
Annotation report		
Tool used	PGAP	
Genes (total)	4,481	
CDS (total)	4,392	
Genes (coding)	4,313	
CDSs (with protein)	4,313	
rRNA	5, 4, 11 (5S, 16S, 23S)	
tRNAs	65	
ncRNAs	4	
CDSs (without protein)	79	
CRISPR arrays	2	
Genome report (BV-BRC)		
Coarse consistency (%)	97.5	
Fine consistency (%)	94.8	
Completeness (%)	99.8	
Contamination (%)	1.7	
Special gene (BV-BRC)		
Antibiotic resistance	PATRIC	34
	NDARO	4
	CARD	6
Virulence factor	PATRIC_VF	2
	VFDB	25
	Victors	11
Transporter	TCDB	17
Drug target	DrugBank	7
	TTD	2

## Data Availability

This Whole Genome Shotgun project has been deposited in GenBank under accession no. JBLYEK000000000.1. Raw reads are available in the SRA under SRR35227819. Associated records include BioSample SAMN47177161 and BioProject PRJNA1230369. The assembly is listed as ASM4851455v1. The 16S rRNA gene is PV113231.1, and the genome is also available in BV-BRC under ID 71452.104.
